# Behavioral Phenotypes of Omani Children with Autism Spectrum Disorder

**DOI:** 10.3390/bs16101955

**Published:** 2026-10-20

**Authors:** Ahmed Al Saidi, Moon Fai Chan, Watfa Al-Mamari, Ahmed B. Idris, Sara Al Shirawi, Zamzam Al Hashmi

**Affiliations:** 1 Developmental Pediatric Unit, Department of Child Health, Sultan Qaboos University Hospital, University Medical City, Al-Khoudh, P.O. Box 112, Muscat 123, Oman; watfa@squ.edu.om (W.A.-M.); a.idris1@squ.edu.om (A.B.I.); 2 Department of Child Health, College of Medicine & Health Sciences, Sultan Qaboos University, Al-Khoudh, P.O. Box 50, Muscat 123, Oman; 3 Department of Family Medicine & Public Health, College of Medicine & Health Sciences, Sultan Qaboos University, Al-Khoudh, Muscat 123, Oman; moonf@squ.edu.om; 4 School of Medicine, Royal College of Surgeons in Ireland, 123 St Stephen’s Green, D02 YN77 Dublin, Ireland; saraalshirawi23@rcsi.com; 5 Deanship of Undergraduate Studies, College of Medicine & Health Sciences, Sultan Qaboos University, Al-Khoudh, P.O. Box 35, Muscat 123, Oman; s144295@student.squ.edu.om

**Keywords:** autism spectrum disorder, behavioral phenotype, cluster analysis, restricted and repetitive behaviors, social dimensions, DARB, SSDS, Oman, consanguinity, verbal status

## Abstract

**Background:** Autism spectrum disorder (ASD) is characterized by substantial phenotypic heterogeneity, which can complicate clinical formulation and intervention planning. Despite increasing epidemiological research on ASD in the Sultanate of Oman, systematic behavioral variation among Omani children with ASD has not been reported in the published literature. This study aimed to explore patterns of behavioral profiles in an Omani clinical cohort using multidimensional measures of repetitive behavior and social functioning. **Methods:** We conducted a cross-sectional study of 205 children with DSM-5-confirmed ASD (mean age 5.3 ± 2.6 years; 80.5% male) attending specialist services in Muscat, Oman. Parents completed the Dimensional Assessment of Repetitive Behavior (DARB) and the Stanford Social Dimensions Scale (SSDS), two multidimensional parent-report instruments. Two-step cluster analysis was used to explore clusters representing a continuum of behavioral characteristics. Verbal and non-verbal children were also compared across DARB and SSDS dimensions. **Results:** A two-cluster solution (silhouette coefficient = 0.40, indicating weak-to-fair separation) described a severity gradient: a higher-severity cluster (n = 59, 28.8%) with elevated scores across all eight DARB subdomains and lower scores across all five SSDS dimensions, and a lower-severity cluster (n = 146, 71.2%). Clusters did not differ significantly on the demographic variables examined, including consanguinity (*p* = 0.489). Among verbal children (n = 126), social motivation, affiliation, expressive communication, and social recognition scores were higher than among non-verbal children (n = 79). Non-verbal children demonstrated greater sensory–motor stereotypy. **Conclusions:** This exploratory study provides an initial characterization of behavioral variation among children with ASD in an Omani clinical cohort. The clustering results suggest a continuum of behavioral characteristics, together with differences associated with verbal status, highlights clinically meaningful heterogeneity within the Omani ASD population. These findings are hypothesis-generating and may help inform future research on individualized assessment and intervention in Oman and the wider Arab Gulf region.

## 1. Introduction

Autism spectrum disorder (ASD) is a lifelong neurodevelopmental condition defined by two core symptom domains: persistent deficits in social communication and social interaction, and restricted, repetitive patterns of behavior, interests, and activities (American Psychiatric Association, 2022). Within this unified diagnostic category exists a clinical population of extraordinary phenotypic diversity, encompassing individuals who differ not only in the severity but also in the configuration of social and behavioral features, their developmental trajectories, cognitive profiles, and treatment responsiveness (Lord et al., 2000; Warrier et al., 2022). This heterogeneity represents one of the most pressing unresolved challenges in ASD science, because effective early intervention requires individualized clinical formulation rather than generic, severity-based treatment plans. Data-driven approaches that identify naturalistic behavioral subgroups—referred to broadly as phenotyping—have emerged as a principled strategy for addressing this challenge, with the additional potential to link behavioral profiles to genetic, neurobiological, and environmental mechanisms (Kim et al., 2016; Syriopoulou-Delli & Papaefstathiou, 2020).

In the Sultanate of Oman, the epidemiology of ASD has undergone substantial documentation over the past 15 years, yet behavioral phenotyping of this population remains entirely absent from the published literature. The foundational national prevalence study (Al-Farsi et al., 2011), using DSM-IV-TR criteria and the Childhood Autism Rating Scale, identified 113 cases nationwide yielding a prevalence of 1.4 per 10,000 children, acknowledged by the authors as a substantial underestimate attributable to underdiagnosis and underreporting. The subsequent multicentre study by (Al-Mamari et al., 2019), drawing on records from Sultan Qaboos University Hospital (SQUH), Royal Hospital, and Al-Massarah Hospital and applying DSM-5 criteria across 2012–2018, identified 1705 ASD cases and reported a prevalence of 20.35 per 10,000 (95% CI [19.39, 21.32]), with boys comprising 78.1% of cases and Muscat showing the highest regional prevalence (36.51/10,000). In a parallel case–control study at SQUH, Al-Mamari et al. (2021a) identified advancing maternal age as the dominant parental risk factor for ASD in Oman, with adjusted odds ratios rising monotonically from 3.39 (ages 25–29) to 13.13 (≥40 years), whereas the parallel paternal-age effect was smaller and attenuated to non-significance beyond 45 years (OR = 1.42, 95% CI 0.64–3.15)—a divergence that, given advancing paternal age as the principal demographic proxy for de novo germline mutational burden, points to a genetic architecture weighted toward inherited recessive rather than paternal-age-dependent de novo variation and warrants dedicated exploration. An additional publication from the same group documented intellectual profiles of children with ASD in Oman, highlighting significant within-sample cognitive heterogeneity (Al-Mamari et al., 2021b). Looking prospectively, Al-Mamari et al. (2025a) have projected that the number of Omani children requiring developmental-behavioral pediatric services will increase substantially through 2040, driven by population growth and improved ascertainment, placing acute demands on a service system already operating at capacity.

The 15-fold prevalence increase between 2011 and 2019 requires critical contextualization (Al-Mendalawi, 2020), in a letter to the editor responding to (Al-Mamari et al., 2019), raised a methodological concern that has not been adequately addressed in the subsequent literature: the two estimates employed fundamentally different diagnostic systems—DSM-IV-TR versus DSM-5 reported that the proposed DSM-5 criteria identified only 60.6% of individuals with a DSM-IV-TR pervasive developmental disorder diagnosis (specificity 94.9%), with the lowest sensitivity among individuals with higher IQ and those with Asperger’s Disorder or PDD-NOS. Because the two Omani estimates were derived under diagnostic systems with imperfect concordance, part of the apparent increase may reflect criterion change and expanded ascertainment rather than a change in incidence; the direction and magnitude of this effect in Oman are unknown. Disentangling criterion change from genuine epidemiological change is necessary for accurate policy planning.

A socio-cultural dimension further shapes ASD ascertainment in Oman that quantitative prevalence studies do not address. Oman is a collectivist society in which family cohesion, conformity to group norms, and the preservation of family honour exert strong regulatory pressure on the recognition and disclosure of atypical child development (Al-Adawi, 2017). Behavioral differences that clinicians would classify as ASD-consistent may be assimilated into culturally normalized accounts of child temperament or religious disposition, delaying recognition and referral. Al-Sharbati et al. (2015) documented that Omani mainstream schoolteachers frequently misattribute ASD behavioral features to willfulness or inadequate parenting. Al-Saadoon et al. (2021) reported low utilization of available services among Omani children with special needs, with stigma and concerns regarding family reputation identified as major barriers to accessing support. The consequence is a clinical sample systematically biased toward the severe end of the ASD behavioral spectrum: children whose presentations are sufficiently impairing to compel help-seeking despite cultural disincentives. This ascertainment bias is a structuring condition that must inform interpretation of all clinical ASD research from Oman.

Compounding this context is Oman’s distinctive genetic landscape. Consanguineous marriage is practiced by approximately 50% of the Omani general population, with 39% being first-cousin unions (Al-Kindi et al., 2019; Islam, 2012). In ASD families specifically, consanguinity rates of 59% have been reported (Al-Mamari et al., 2025b). Genome sequencing of 104 Omani ASD families identified 48 novel candidate genes, with 61% of pathogenic variants homozygous—consistent with recessive inheritance—across 53 distinct genes in 41 consanguineous kindreds (Gupta et al., 2024). Whether this distinctive recessive genetic architecture produces a distinctive behavioral phenotype on dimensional clinical instruments is a question that cannot be addressed without the behavioral characterization that this study provides for the first time.

Internationally, cluster-analytic phenotyping of ASD has most consistently identified severity-gradient solutions rather than qualitatively distinct subtypes, with social communication impairment as the primary discriminating dimension (Syriopoulou-Delli & Papaefstathiou, 2020). Georgiades et al. (2013) applied factor mixture modelling to a North American cohort and identified three latent classes differing primarily in social communication and RRB severity. Kim et al. (2016) demonstrated that early behavioral subtyping in toddlers predicted distinct short-term developmental outcomes, underscoring the clinical value of early phenotyping. Song et al. (2022) showed in a preschool cohort that cluster membership on RRB measures was inversely associated with social skills, with sensory–motor stereotypy and insistence on sameness as the principal discriminating variables. None of these studies involved Arab Gulf populations.

The present study applies two validated, multidimensional instruments—the Dimensional Assessment of Repetitive Behavior (Uljarević et al., 2023) and the Stanford Social Dimensions Scale (Phillips et al., 2019)—to a clinical cohort of Omani children with ASD attending Sultan Qaboos University Hospital (SQUH). Three aims were addressed: (a) to identify naturalistic behavioral subgroups using two-step cluster analysis; (b) to characterize those subgroups across the full DARB and SSDS profiles; and (c) to examine the association between verbal status and dimensional behavioral and social outcomes.

## 2. Methods

### 2.1. Participants and Design

This cross-sectional study recruited participants from the clinical registries of the principal ASD diagnostic center in Oman. Participant identification was conducted through the same institutional infrastructure underpinning major epidemiological studies of ASD in Oman, thereby enhancing comparability with previously reported Omani ASD cohorts. Inclusion criteria required an experienced developmental paediatrician confirmed diagnosis of Autism Spectrum Disorder (ASD) according to DSM-5 criteria (American Psychiatric Association, 2022) using gold-standard diagnostic instruments, including the Autism Diagnostic Interview-Revised (ADI-R) and the Autism Diagnostic Observation Schedule, Second Edition (ADOS-2). Ethical approval for this study was obtained from the Medical Research Ethics Committee, College of Medicine and Health Sciences, Sultan Qaboos University (Ref. No. SQU-EC/036/2026; MREC #3873). Both instruments were completed within routine clinical care prior to the study and were extracted retrospectively from existing de-identified clinical records, with no questionnaire administered specifically for research purposes. All data were anonymized prior to analysis and securely stored on password-protected institutional servers accessible only to the principal investigator and authorized research personnel.

Power analysis followed (Dalmaijer et al., 2022) which indicated a minimum of 160 participants to achieve 87% power (α = 0.05, two-cluster detection, separation distance of 3, silhouette coefficient of 0.50). The final sample comprised 205 children (165 male, 40 female; 80.5% male). Mean age was 5.3 years (SD = 2.6; range 1.6–14.0). The male-to-female ratio of approximately 4.1:1 is consistent with that reported in Oman’s multicentre prevalence study (78.1% male) (Al-Mamari et al., 2019) with global epidemiological data (Zeidan et al., 2022). Among the cohort from Oman, consanguinity was reported in 45.4% of families. Based on parental report of functional spoken language use, 126 children (61.5%) were classified as verbal and 79 (38.5%) as non-verbal. No missing data were identified on either instrument.

### 2.2. Measures

Dimensional Assessment of Repetitive Behavior (DARB) (Uljarević et al., 2023) is a 99-item parent/caregiver questionnaire (84 core items; 15 supplemental restricted-interest items) designed to provide dimensional assessment of eight empirically derived RRB subdomains: (1) Repetitive Sensory–Motor Behaviors (20 items; e.g., “Does your child rock body, either when standing or sitting?”); (2) Insistence on Sameness (20 items; e.g., “Does your child insist that particular activities have to take place at the exact same time of the day regardless of the situation or context?”); (3) Self-Injurious Behaviors (9 items); (4) Sensory Sensitivity (9 items; e.g., “Does your child react more strongly than other people to loud or sudden noises?”); (5) Unusual Interests (8 items; e.g., “Does your child have a fascination with dates and/or time?”); (6) Obsessive–Compulsive Behaviors (9 items); (7) Repetitive Language (8 items; e.g., “Does your child immediately repeat/echo what you or others have said?”); and (8) Restricted Interests (15 items). Each item is rated on a five-point Likert scale (1 = Not at all a problem to 5 = Very serious). In our sample, the DARB have a good internal reliability overall (Cronbach’s alpha: 0.980) and its subdomains (Repetitive Sensory–Motor Behaviors: 0.956; Insistence on Sameness: 0.957; Self-Injurious Behaviors: 0.861; Sensory Sensitivity: 0.882; Unusual Interests: 0.828; Obsessive–Compulsive Behaviors: 0.891; Restricted Interests: 0.920).

Stanford Social Dimensions Scale (SSDS) (Phillips et al., 2019) is a 71-item parent/caregiver questionnaire measuring five social process dimensions: (1) Social Motivation (14 items; e.g., “In a social situation, attempts to play with other children instead of avoiding the group”); (2) Social Affiliation (8 items; e.g., “Will try to get my attention or interact with me, without being reminded”); (3) Expressive Social Communication (7 items; e.g., “Vocalizes and makes eye contact when making a request”); (4) Social Recognition (7 items; e.g., “Reads subtle emotions in others through their facial expressions”); and (5) Unusual Approach Behaviors (4 items; e.g., “Begins interactions in ways that seem unusual to others”). The first 58 items are appropriate for any child regardless of language level and constitute the validated factor-analytic subscales. Items are rated 1–5 (1 = Never true to 5 = Always true). Higher scores reflect better social functioning. In our sample, the SSDS showed good internal reliability overall (Cronbach’s alpha: 0.947) and across subdimensions (Social Motivation: 0.919; Social Affiliation: 0.858; Expressive Social Communication: 0.817; Social Recognition: 0.903; Unusual Approach Behaviours: 0.676).

#### Translation and Cultural Adaptation of the Instruments

As neither the DARB nor the SSDS had a validated Arabic version, both measures were adapted into Arabic in line with established recommendations for the cross-cultural adaptation of questionnaire-based instruments (Beaton et al., 2000; Wild et al., 2005). The process began with forward translation, in which two bilingual translators independently rendered the English originals into Arabic. Their versions were then compared, and any differences were discussed until agreement was reached on a single draft. Next, a third bilingual translator, who had no access to the source instruments, translated this draft back into English. The research team examined the source, Arabic, and back-translated versions side by side, resolving inconsistencies with the aim of achieving equivalence in meaning rather than word-for-word correspondence.

The draft Arabic versions were then reviewed by a multidisciplinary panel of experts in clinical psychology, behavioral psychology, developmental-behavioral pediatrics, and psychometrics, all familiar with clinical practice in Oman. Panel members rated each item for linguistic clarity, cultural relevance, and conceptual equivalence to the source. Their input led to small wording adjustments that made the items easier to understand, while the underlying constructs, response options, and scoring rules of the original instruments were retained unchanged.

Finally, the Arabic versions were piloted with a small sample of Omani parents resembling the intended study population, to examine how clear and understandable the items were and how long the questionnaires took to complete. Pilot participants suggested only minor changes to phrasing; the content and scoring of the instruments were not altered. The internal consistency coefficients obtained in the current sample provide additional support for the reliability of the Arabic versions used in this study.

Verbal status was classified by parental binary report of whether the child regularly used spoken language communicatively (verbal) or lacked functional spoken communication (non-verbal). Parents received standardized instructions clarifying that ‘verbal’ required purposive communicative speech—not echolalia or isolated vocalisation—to minimise misclassification at the functional boundary. This pragmatic classification reflects expressive functional language only and was used because of its direct relevance to intervention pathway selection; it does not capture receptive language or the heterogeneity of minimally verbal presentations (Tager-Flusberg & Kasari, 2013).

### 2.3. Statistical Analysis

Descriptive statistics were computed for all demographic and outcome variables. We implemented two-step cluster analysis in IBM SPSS Statistics v25.0, using all DARB and SSDS subscale scores (continuous) and demographic variables (categorical) as clustering inputs. This algorithm accommodates mixed continuous-categorical data structures without requiring a pre-specified cluster number, selecting the cluster count by minimising the Bayesian Information Criterion (BIC) (Dalmaijer et al., 2022). The log-likelihood distance measure was applied. Solution quality was evaluated using the average silhouette coefficient: values ≥ 0.50 indicate strong cluster structure, 0.20–0.49 indicate fair-to-weak structure, and <0.20 indicate absent structure (Kaufman & Rousseeuw, 1990; Peiró et al., 2025). Between-cluster and verbal-status comparisons on continuous variables used Mann–Whitney U tests; categorical comparisons used Pearson chi-square or Fisher’s exact test. We used α = 0.05 as the significance threshold, but because we examined multiple comparisons, we applied a Benjamini–Hochberg adjusted *p*-value.

## 3. Results

### 3.1. Demographic and Psychological Profile

A total of 205 children were included in the analysis. In Table 1, the average age is 5.3 (SD = 2.6) years, and boys comprised nearly 80% of the cohort (n = 165). The average ages of the father and mother are 39.6 and 36.7, respectively, and the average number of siblings in the family is 2.5. While both fathers (n = 105) and mothers (n = 198) had higher levels of education, more than half had a primary/high school education. While the majority of their fathers (n = 198) are working, the majority of their mothers (n = 84) are not working, which was around 40%. The psychological outcomes are summary in Table 2, in the 8 types of restricted and repetitive behaviors and interests (RRB) of DARB, the mean ± SD of the Repetitive Sensory Motor Behaviors, Insistence on Sameness Domain, Self-Injurious Behaviors, Sensory Sensitivity, Unusual Interests, Obsessive Compulsive Behaviors, Repetitive Language, and Restricted Interests was 15.3 ± 16.2, 15.4.7 ± 16.9, 2.8 ± 4.7, 7.8 ± 7.6, 4.9 ± 5.9, 4.5 ± 6.3, 7.8 ± 7.9, and 10.2 ± 10.2, respectively. In the 5 sub-social interaction of SSDS scores, the mean ± SD of Social motivation, Social affiliation, Expressive social communication, social recognition, and Unusual apperception was 44.5 ± 13.3, 25.1 ± 7.7, 24.0 ± 6.3, 21.5 ± 7.7, and 13.5 ± 3.7, respectively.

### 3.2. Comparison of Demographic Characteristics and Psychological Outcomes by Clusters

The two-step cluster analysis yielded a two-cluster solution (silhouette coefficient = 0.40; BIC = 1837.58; Ratio of Distance Measure = 2.568), partitioning the sample into Cluster 1 (n = 59; 28.8%) and Cluster 2 (n = 146; 71.2%). We also compared with 3 clusters (BIC = 1789.33; Ratio of Distance Measure = 1.656) and 4 clusters (BIC = 1817.12; Ratio of Distance Measure = 1.244), while the 2 clusters had the highest BIC and Ratio of Distance Measure. Shared patterns across demographic and psychological indicators drove grouping. Demographically, the two clusters were largely indistinguishable (Table 1). In contrast, psychosocial profiles diverged clearly (Table 2).

**Figure 1:**
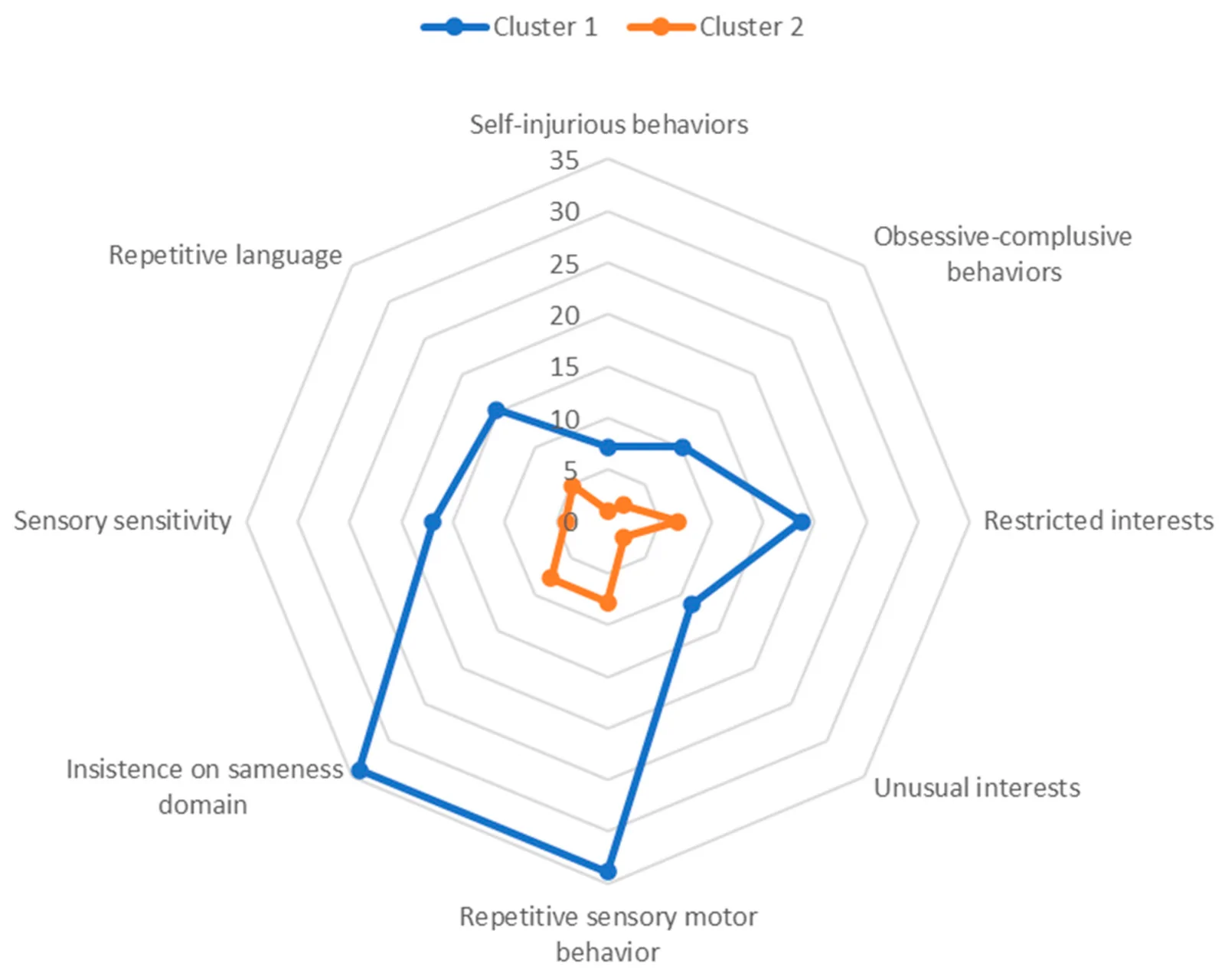
Sub-domains on the Dimensional Assessment of Repetitive Behavior.

As illustrated in Figure 1, participants in Cluster 1 consistently displayed markedly elevated scores across multiple behavioral domains, including repetitive sensory–motor behaviors (33.9 ± 14.0), insistence on sameness (34.4 ± 17.7), self-injurious behaviors (7.2 ± 6.4), sensory sensitivity (16.9 ± 6.9), unusual interests (11.4 ± 6.5), obsessive–compulsive features (10.2 ± 8.3), repetitive language (15.2 ± 8.8), and restricted interests (18.7 ± 12.3), all significantly higher than those observed in Cluster 2 (repetitive sensory motor behaviors: 7.8 ± 9.6; *p* < 0.001; insistence on sameness domain: 7.8 ± 8.5, *p* < 0.001; self-injurious behaviors: 1.0 ± 1.9, *p* < 0.001; sensory sensitivity: 4.1 ± 3.8, *p* < 0.001; unusual interests: 2.2 ± 2.6, *p* < 0.001; obsessive–compulsive behaviors: 2.2 ± 3.2, *p* < 0.001; repetitive language: 4.8 ± 5.2, *p* < 0.001, and restricted interests: 6.8 ± 6.6, *p* < 0.001).

**Figure 2:**
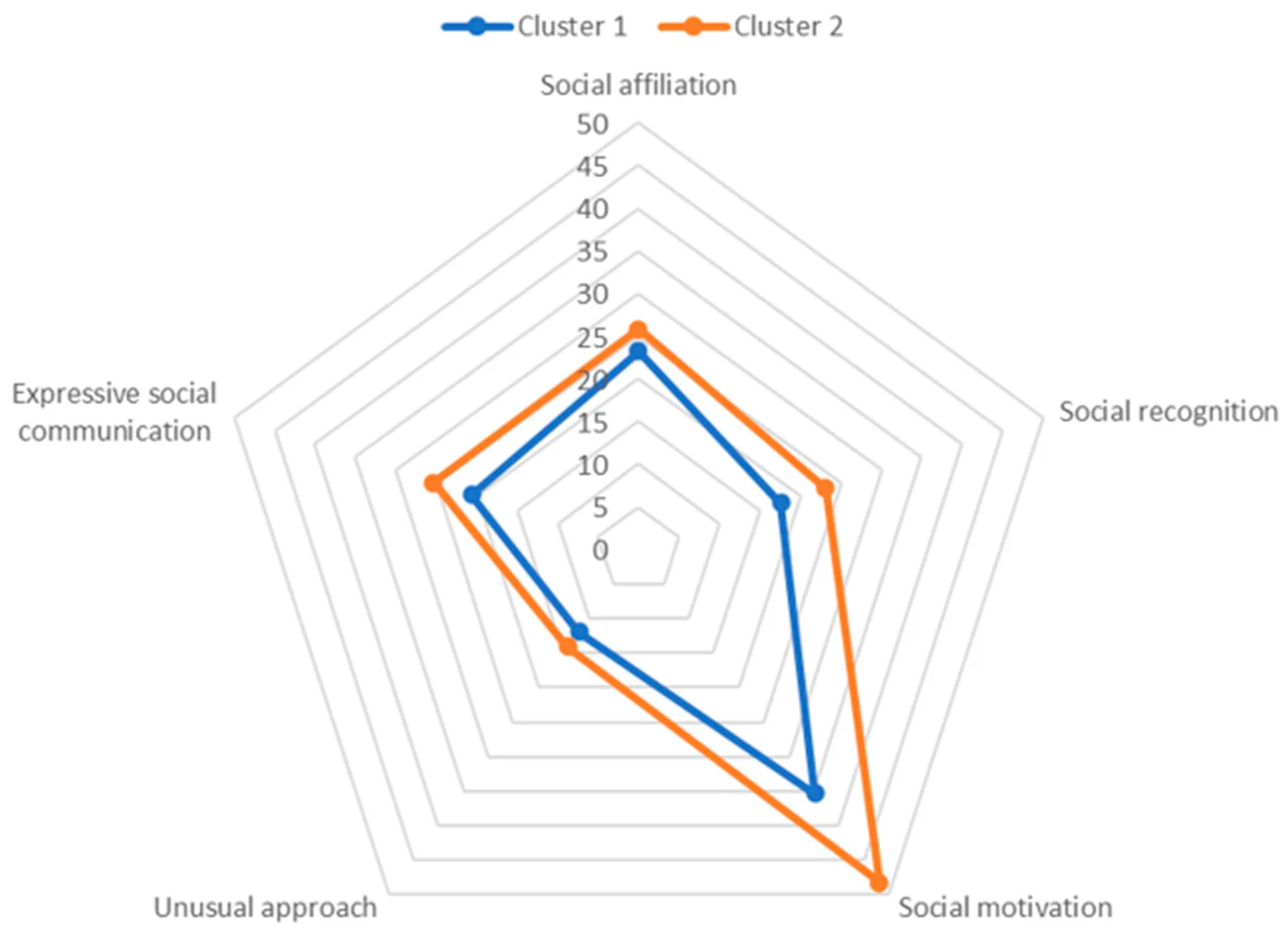
Sub-domains on the Standford Social Dimensions Scale.

By contrast, Figure 2 shows that Cluster 1 exhibited consistently lower scores across social domains measured by the SSDS. Specifically, social motivation (35.3 ± 9.9), social affiliation (23.2 ± 7.4), expressive social communication (20.6 ± 5.8), social recognition (17.6 ± 6.7), and unusual approach behaviors (11.9 ± 4.0) were all reduced relative to Cluster 2, where substantially higher scores were observed (social motivation: 48.2 ± 12.7, *p* < 0.001; social affiliation: 25.8 ± 7.7, *p* = 0.014; expressive social communication: 25.3 ± 5.9, *p* < 0.001; social recognition: 23.1 ± 7.6, *p* < 0.001; and unusual approach: 14.1 ± 3.4, *p* < 0.001). Taken together, these findings suggest a profile in Cluster 1 characterized by heightened repetitive and restrictive behaviors alongside comparatively diminished social functioning.

### 3.3. Comparison of Psychological Outcomes by Parental Report of the Child’s Verbal Status

As shown in Table 3, non-verbal children scored higher than verbal children on repetitive sensory–motor behaviors (20.1 ± 18.0 vs. 12.3 ± 14.2; adjusted *p* < 0.001). Repetitive language was descriptively higher in verbal children (8.3 ± 7.5 vs. 7.0 ± 8.6), but this difference did not survive Benjamini–Hochberg correction (unadjusted *p* = 0.034; adjusted *p* = 0.064). The remaining six DARB domains did not differ significantly between the groups.

A similar divergence emerged in the SSDS domains. Verbal children showed elevated scores in social motivation (47.2 ± 13.0), social affiliation (27.6 ± 6.9), expressive social communication (25.3 ± 6.0), and social recognition (23.7 ± 7.3) than non-verbal children (social motivation: 40.1 ± 12.8, *p* < 0.001; social affiliation: 21.0 ± 7.2, *p* < 0.001; expressive social communication: 21.9 ± 6.2, *p* < 0.001; and social recognition: 18.0 ± 7.1, *p* < 0.001). Unusual approach scores did not differ significantly by verbal status (verbal: 13.0 ± 3.6; non-verbal: 14.2 ± 3.9; unadjusted *p* = 0.066; adjusted *p* = 0.146). Because all SSDS subscales, including Unusual approach, are scored so that higher values indicate better functioning, the non-verbal group’s marginally higher mean does not indicate more unusual approach behavior.

## 4. Discussion

This study presents the first exploratory, dimensional behavioral profiling of children with ASD in Oman, using the DARB and SSDS to characterise a clinical cohort of 205 children across the full multidimensional structure of both ASD core symptom domains. An exploratory two-cluster solution described a severity gradient from a higher- to a lower-severity behavioral profile; cluster membership was not significantly associated with the demographic variables examined, including consanguinity; and verbal status was associated with lower sensory–motor stereotypy and higher scores on four of five SSDS dimensions. Three substantive discussion points follow.

### 4.1. The Cluster Structure: Internal Architecture and International Context

The present findings from the Omani cohort are consistent with international evidence demonstrating the emergence of high-severity and moderate-severity behavioral subgroups, thereby replicating one of the most consistently reported patterns in the ASD phenotypic clustering literature (Georgiades et al., 2013; Kim et al., 2016; Syriopoulou-Delli & Papaefstathiou, 2020).

What distinguishes the current findings is the simultaneous elevation of all eight DARB subdomains in Cluster 1—encompassing both lower-order sensorimotor behaviors (repetitive motor behaviors, sensory sensitivity, self-injury) and higher-order cognitive-regulatory behaviors (insistence on sameness, obsessive–compulsive features, restricted interests). Prior factor-analytic work has consistently identified at least two semi-independent RRB factors—a repetitive sensorimotor factor and an insistence on sameness factor with distinct developmental trajectories and cognitive correlates (Harrop et al., 2014). The uniform elevation of both classes in Cluster 1 argues for a globally dysregulated phenotype rather than a selective RRB subtype, consistent with the broadly affected profile identified in genomic-behavioral clustering work linking greater polygenic burden to multidomain severity (Leisman & Melillo, 2025; Zahiri et al., 2024).

Concurrently, the reduced Social Motivation in Cluster 1 (M = 35.3) relative to Cluster 2 (M = 48.2) is theoretically noteworthy. Social motivation—the hedonic drive to engage with other people—is proposed as a primary mechanism in ASD, with attenuated social reward salience driving downstream deficits in social learning and communication (Chevallier et al., 2012). That reduced social motivation co-occurs with maximum RRB severity across all eight DARB subdomains in Cluster 1 is consistent with a shared regulatory deficit across both core symptom domains, potentially reflecting attenuated mesolimbic reward system function rather than two independent pathological processes that happen to co-occur (Bertelsen et al., 2021; Blum et al., 2024; Supekar et al., 2018).

The realised silhouette coefficient of 0.40, while within the fair-to-weak range, fell below the value of 0.50 assumed in the a priori planning scenario, indicating weaker separation than anticipated. The case-order stability analysis [supported/only partially supported] the two-cluster solution ([k] of [20] re-runs; mean adjusted Rand index [x]). Because the two clusters are ordered on severity across all 13 clustering variables and cluster membership correlates strongly with total scores, the partition operationalizes a severity gradient rather than a qualitatively distinct subtype. A coefficient in this range further confirms that the cluster boundaries are gradients rather than discontinuities—the pattern expected for a dimensionally distributed condition. Beyond these methodological considerations, the modest separation may also carry a substantive population-specific component: the recessive-weighted and markedly heterogeneous genetic architecture of Omani ASD, in which numerous distinct homozygous variants across many genes converge on phenotypically overlapping presentations (Gupta et al., 2024), could plausibly attenuate the discreteness of behaviorally defined subgroups and yield a more continuously distributed phenotype than that observed in the outbred Western and East Asian cohorts from which prior clustering solutions were derived (Georgiades et al., 2013; Kim et al., 2016; Song et al., 2022). This possibility cannot presently be adjudicated; however, as no behavioral phenotyping data from any Arab or Gulf population exist against which the present separation can be benchmarked; the current cohort therefore constitutes the first regional reference point rather than a deviation from an established norm. The two-cluster solution should accordingly be understood as a provisional descriptive phenotyping scaffold rather than a claim about ASD natural kinds, and requires independent replication in larger samples incorporating objective observational data (e.g., Autism Diagnostic Observation Schedule, Second Edition calibrated severity scores) and formal cognitive assessment.

### 4.2. Consanguinity and the Genotype–Behavioral Phenotype Dissociation

The absence of an association between consanguinity and cluster membership (*p* = 0.489) warrants consideration. As no genetic data were collected in this study, this finding cannot be explained directly; however, one plausible interpretation arises from published genomic evidence showing a highly heterogeneous, recessive-weighted genetic architecture in Omani ASD, with pathogenic homozygous variants distributed across many different genes (Gupta et al., 2024). Under such an architecture, children from consanguineous families may carry entirely different variants yet present with overlapping behavioral profiles on parent-report measures (Morrow et al., 2008). Binary consanguinity reflects only the family-level risk of homozygosity, not the specific variants a child carries, and may therefore be too coarse to detect genotype-related behavioral differences.

This interpretation gains support from pharmacological evidence within Oman. found that Omani children with a family history of ASD—a proxy for inherited, likely recessive-variant ASD—had dramatically lower risperidone response rates than those without family history demonstrating that genotypically distinct ASD subtypes produce pharmacologically distinguishable behavioral profiles even when dimensional symptom measures do not differentiate them (Al-Huseini et al., 2022). One possible reading of the present null finding is that binary consanguinity lacks the resolution to detect genotype-related behavioral differences; the present data cannot distinguish this from a true absence of association, and non-significance should not be read as equivalence. Future genotype–phenotype analyses should incorporate genomic homozygosity metrics as continuous measures rather than binary consanguinity self-report.

### 4.3. Verbal Status and the Modular Behavioral Signature

After multiplicity correction, one DARB subdomain, Repetitive Sensory–Motor Behaviors, differed by verbal status. Non-verbal children showed greater Repetitive Sensory–Motor Behaviors (adjusted *p* < 0.001), consistent with the hypothesis that stereotyped behavioral output is distributed across available output channels: when verbal channels are unavailable, motor stereotypy serves as the predominant self-regulatory modality (Patten et al., 2013). Repetitive Language was descriptively higher in verbal children (adjusted *p* = 0.064), as would be expected since verbal stereotypy (echolalia, scripted speech, perseverative questioning) presupposes functional verbal capacity; however, this difference did not survive correction and we do not interpret it further. We also note that a significant difference in one domain alongside non-significant differences in others does not establish that the associations differ in magnitude; this would require a direct test of the interaction, which we did not undertake. With that caveat, the pattern is compatible with the hypothesis that expressive language status modulates the channel of stereotyped output rather than its overall regulatory burden (Kasari et al., 2013; Lanzarini et al., 2021; Masjedi et al., 2025; Tager-Flusberg & Kasari, 2013).

The SSDS findings indicate that verbal capacity is associated with superior performance on four of five social dimensions. This association may reflect the mutual developmental dependence of language and social motivation: children who develop functional speech are those for whom social reward is sufficiently salient to drive language acquisition in social contexts, and their verbal capacity in turn expands the range of social strategies available, producing the superior SSDS profiles observed. Unusual Approach scores did not differ significantly by verbal status; on this reverse-scored subscale the non-verbal group’s marginally higher mean would, if anything, indicate fewer unusual approach behaviors, and we draw no inference from it. Verbal status may nonetheless function as a developmental moderator that shapes both the expression of restricted and repetitive behaviors and broader social presentation. Within this context, family and community responses may either reinforce or discourage certain behaviors, including adaptive self-help skills. In some cases, parental beliefs that language and social communication will improve naturally with age may contribute to delayed intervention-seeking, particularly when developmental differences are interpreted as temporary or part of the child’s expected maturation process.

### 4.4. Limitations and Future Directions

Several limitations constrain interpretation. The cross-sectional design precludes causal inference and cannot establish the developmental stability of cluster membership across time—a particular limitation given that RRB trajectories differ systematically by subdomain across development (Harrop et al., 2014). The silhouette coefficient of 0.40 confirms a weak-to-fair cluster solution requiring independent replication. In addition, the two-step method assumes that continuous variables are normally distributed, categorical variables follow a multinomial distribution, and all variables are mutually independent within the model. However, these are model assumptions, not a requirement that every continuous variable in the dataset be normal before clustering can proceed. The key distinction is that non-normality does not automatically invalidate the analysis. Still, it can affect the accuracy and stability of the resulting clusters. The case-order stability analysis reported above [supports/only partially supports] the two-cluster solution, but stability is not validity: the partition has not been tested against external criteria, and the between-cluster differences in Table 2 cannot serve that purpose because those variables defined the clusters. Verbal status was determined by binary parental report rather than standardised language assessment, limiting precision at the functional speech boundary. This classification does not capture receptive language or distinguish children with a few words from those with limited phrase speech, and, given the young age of the cohort, some non-verbal children may be preverbal rather than minimally verbal. Future studies should use standardized, continuous measures of expressive and receptive language. The sample was drawn exclusively from Muscat-based tertiary center and likely overrepresents the severe end of the ASD phenotypic distribution, given the cultural and systemic barriers to diagnosis documented by Al-Sharbati et al. (2015) and Al-Saadoon et al. (2021). In the absence of cognitive assessment data, the observed severity gradient should be interpreted as potentially reflecting autism-specific characteristics, general developmental level, or an interplay of both. ADOS-2 was used for diagnostic confirmation, but its scores were not available for analysis. The DARB and SSDS were validated primarily in English-speaking samples; formal Arabic-language psychometric validation in Omani populations has not yet been published.

Future research should integrate the genomic data with DARB and SSDS profiles to test whether behavioral clusters correspond to genotypic subgroups. Longitudinal follow-up would determine whether early cluster membership predicts developmental trajectory and intervention response. As the national screening programme matures and ascertainment expands to milder presentations, the phenotypic composition of clinical samples will shift; monitoring this shift constitutes a natural experiment in behavioral epidemiology that the present baseline enables.

## 5. Conclusions

This study provides the first dimensional behavioral profiling of children with ASD in Oman using the DARB and SSDS. Exploratory clustering described two groups along a severity gradient—a higher-severity cluster (28.8% of cases) with elevated scores across all eight RRB subdomains and lower scores across all five social dimensions, and a lower-severity cluster (71.2%)—which did not differ significantly on the demographic variables examined, including consanguinity; this does not establish independence. Because cognitive assessment data were unavailable, the gradient may partly index general developmental level rather than autism-specific severity, and the clusters should be treated as hypothesis-generating observations rather than established subgroups. Given the heterogeneous genetic architecture reported in Omani ASD, binary consanguinity may be too coarse to capture genotype-related behavioral differences, a hypothesis that future genotype–phenotype studies should test. Verbal status was associated with lower sensory–motor stereotypy and higher scores on four SSDS social dimensions. The findings must be interpreted against the ascertainment bias inherent in Omani clinical ASD samples, which the combined operation of collectivist cultural norms, stigma-driven concealment, and limited diagnostic infrastructure skews toward the severe end of the population distribution. If replicated with cognitive and observational measures, these results could provide a behavioral baseline for the planning of the expanded ASD service capacity identified as needed through 2040, and a starting point for genotype–phenotype correlation studies in this genetically distinctive population.

## Figures and Tables

**Table 1:** Sample demographic data and comparison by clusters.

		Cluster		Test ~	
Characteristics	Total (n = 205) n (%)	1 (n = 59, 28.8%) n (%)	2 (n = 146, 71.2%) n (%)	Statistics	*Adj p*-Value
Gender					
Boy	165 (80.5)	48 (81.4)	117 (80.1)	0.040 ~	0.892
Girl	40 (19.5)	11 (18.6)	29 (19.9)		
Child age (years):					
Median [Range]	5.0 [1.6–14.0]	4.0 [1.8–13.0]	5.0 [1.6–14.0]	0.635 #	0.630
Mean ± SD	5.3 ± 2.6	5.0 ± 2.2	5.4 ± 2.7		
Consanguinity					
Yes	93 (45.4)	29 (49.2)	64 (43.8)	0.479 ~	0.629
No	112 (54.6)	30 (50.8)	82 (56.2)		
Education (Mother)					
Primary/High school	108 (52.7)	32 (54.2)	76 (52.1)	0.080 ~	0.614
University	97 (47.3)	27 (45.8)	70 (47.9)		
Education (Father)					
Primary/High school	105 (51.2)	34 (57.6)	71 (48.6)	1.361 ~	0.380
University	100 (48.8)	25 (42.4)	75 (51.4)		
Working status (Father)					
Retired/Unemployed	7 (3.4)	2 (3.4)	5 (3.4)	<0.001 ^	0.999
Employed	198 (96.6)	57 (96.6)	141 (96.6)		
Working status (Mother)					
Retired/Unemployed/Housewife	121 (59.0)	37 (62.7)	84 (57.5)	0.466 ~	0.495
Employed	84 (41.0)	22 (37.3)	62 (42.5)		
Father’s age (years)					
Median [Range]	38.0 [29.0–65.0]	36.0 [30.0–58.0]	39.0 [29.0–65.0]	1.514 #	0.223
Mean ± SD	39.6 ± 7.0	38.4 ± 6.1	40.1 ± 7.2		
Mother’s age (years)					
Median [Range]	36.0 [23.0–53.0]	35.0 [24.0–51.0]	36.5 [23.0–53.0]	0.524 #	0.698
Mean ± SD	36.7 ± 6.3	36.3 ± 5.7	36.8 ± 6.6		
Number of siblings					
Median [Range]	2.0 [0.0–9.0]	2.0 [0.0–7.0]	2.0 [0.0–9.0]	0.385 #	0.788
Mean ± SD	2.5 ± 1.6	2.5 ± 1.5	2.5 ± 1.6		

^, Fisher exact test; #, Mann–Whitey U test (|z|); ~, c^2^ test; SD, standard deviation; Adj *p*-value, Benjamini–Hochberg correction.

**Table 2:** Sample psychological data and comparison by clusters.

		Cluster		Test #		
Psychological	Total (n = 205) n (%)	1 (n = 59) n (%)	2 (n = 146) n (%)	|z| Statistics	*Adj p*-Value	Effect Sizes
DARB						
Repetitive sensory motor behavior						
Median [Range]	9.0 [0.0–69.0]	35.0 [6.0–69.0]	4.0 [0.0–53.0]	9.881	<0.001 **	0.690
Mean ± SD	15.3 ± 16.2	33.9 ± 14.0	7.8 ± 9.6			
Insistence on sameness domain						
Median [Range]	10.0 [0.0–80.0]	31.0 [1.0–80.0]	5.0 [0.0–39.0]	9.545	<0.001 **	0.667
Mean ± SD	15.4.7 ± 16.9	34.4 ± 17.7	7.8 ± 8.5			
Self-injurious behaviors						
Median [Range]	1.0 [0.0–25.0]	6.0 [0.0–25.0]	0.0 [0.0–13.0]	8.701	<0.001 **	0.608
Mean ± SD	2.8 ± 4.7	7.2 ± 6.4	1.0 ± 1.9			
Sensory Sensitivity						
Median [Range]	5.0 [0.0–34.0]	16.0 [5.0–34.0]	3.0 [0.0–19.0]	10.352	<0.001 **	0.723
Mean ± SD	7.8 ± 7.6	16.9 ± 6.9	4.1 ± 3.8			
Unusual interests						
Median [Range]	3.0 [0.0–31.0]	11.0 [0.0–31.0]	1.0 [0.0–13.0]	9.508	<0.001 **	0.664
Mean ± SD	4.9 ± 5.9	11.4 ± 6.5	2.2 ± 2.6			
Obsessive–compulsive behaviors						
Median [Range]	2.0 [0.0–36.0]	9.0 [0.0–36.0]	1.0 [0.0–16.0]	7.672	<0.001**	0.536
Mean ± SD	4.5 ± 6.3	10.2 ± 8.3	2.2 ± 3.2			
Repetitive language						
Median [Range]	6.0 [0.0–32.0]	14.0 [0.0–32.0]	3.5 [0.0–25.0]	7.953	<0.001 **	0.555
Mean ± SD	7.8 ± 7.9	15.2 ± 8.8	4.8 ± 5.2			
Restricted interests						
Median [Range]	8.0 [0.0–64.0]	17.0 [0.0–64.0]	5.0 [0.0–31.0]	7.093	<0.001 **	0.495
Mean ± SD	10.2 ± 10.2	18.7 ± 12.3	6.8 ± 6.6			
SSDS						
Social motivation						
Median [Range]	44.0 [14.0–70.0]	35.0 [14.0–61.0]	50.0 [19.0–70.0]	6.168	<0.001 **	0.431
Mean ± SD	44.5 ± 13.3	35.3 ± 9.9	48.2 ± 12.7			
Social affiliation						
Median [Range]	26.0 [8.0–40.0]	23.0 [9.0–40.0]	27.0 [8.0–40.0]	2.459	0.028 *	0.172
Mean ± SD	25.1 ± 7.7	23.2 ± 7.4	25.8 ± 7.7			
Expressive social communication						
Median [Range]	24.0 [7.0–35.0]	21.0 [7.0–35.0]	26.0 [7.0–35.0]	5.128	<0.001 **	0.358
Mean ± SD	24.0 ± 6.3	20.6 ± 5.8	25.3 ± 5.9			
Social recognition						
Median [Range]	21.0 [7.0–35.0]	16.0 [7.0–35.0]	24.0 [7.0–35.0]	4.608	<0.001 **	0.322
Mean ± SD	21.5 ± 7.7	17.6 ± 6.7	23.1 ± 7.6			
Unusual approach						
Median [Range]	13.0 [4.0–20.0]	11.0 [4.0–20.0]	14.0 [5.0–20.0]	3.928	<0.001 **	0.274
Mean ± SD	13.5 ± 3.7	11.9 ± 4.0	14.1 ± 3.4			

#, Mann–Whitey U test; SD, standard deviation; *, Adj *p*-value, Benjamini–Hochberg correction sig. *p* < 0.05; **, Sig. at *p* < 0.01.

**Table 3:** Sample psychological data and comparison by child’s verbal and non-verbal behaviors.

Psychological	Verbal		Test #		
	No (n = 79) n (%)	Yes (n = 126) n (%)	|z| Statistics	*Adj. p*-Value	Effect Sizes
DARB					
Repetitive sensory motor behavior					
Median [Range]	13.0 [0.0–69.0]	6.0 [0.0–59.0]	3.712	<0.001 **	0.259
Mean ± SD	20.1 ± 18.0	12.3 ± 14.2			
Insistence on sameness domain					
Median [Range]	10.0 [0.0–80.0]	10.0 [0.0–73.0]	0.161	0.897	0.011
Mean ± SD	16.2 ± 17.9	15.0 ± 16.4			
Self-injurious behaviors					
Median [Range]	0.0 [0.0–25.0]	1.0 [0.0–25.0]	0.780	0.580	0.054
Mean ± SD	2.7 ± 5.0	2.8 ± 4.6			
Sensory Sensitivity					
Median [Range]	6.0 [0.0–34.0]	5.0 [0.0–34.0]	0.856	0.564	0.060
Mean ± SD	8.6 ± 8.4	7.3 ± 7.0			
Unusual interests					
Median [Range]	3.0 [0.0–31.0]	2.5 [0.0–24.0]	0.814	0.576	0.057
Mean ± SD	5.6 ± 6.7	4.4 ± 5.2			
Obsessive–compulsive behaviors					
Median [Range]	2.0 [0.0–36.0]	3.0 [0.0–31.0]	0.872	0.575	0.061
Mean ± SD	4.4 ± 6.9	4.6 ± 5.9			
Repetitive language					
Median [Range]	4.0 [0.0–32.0]	6.0 [0.0–32.0]	2.123	0.064	0.148
Mean ± SD	7.0 ± 8.6	8.3 ± 7.5			
Restricted interests					
Median [Range]	6.0 [0.0–64.0]	8.5 [0.0–45.0]	1.423	0.254	0.099
Mean ± SD	9.6 ± 11.2	10.6 ± 9.4			
SSDS					
Social motivation					
Median [Range]	38.0 [14.0–70.0]	48.5 [16.0–70.0]	3.796	<0.001 **	0.265
Mean ± SD	40.1 ± 12.8	47.2 ± 13.0			
Social affiliation					
Median [Range]	20.0 [9.0–37.0]	27.5 [8.0–40.0]	5.819	<0.001 **	0.406
Mean ± SD	21.0 ± 7.2	27.6 ± 6.9			
Expressive social communication					
Median [Range]	21.0 [7.0–34.0]	25.0 [7.0–35.0]	3.957	<0.001 **	0.276
Mean ± SD	21.9 ± 6.2	25.3 ± 6.0			
Social recognition					
Median [Range]	17.0 [7.0–34.0]	24.0 [7.0–35.0]	5.116	<0.001 **	0.357
Mean ± SD	18.0 ± 7.1	23.7 ± 7.3			
Unusual approach					
Median [Range]	15.0 [6.0–20.0]	13.0 [4.0–20.0]	2.088	0.066	0.146
Mean ± SD	14.2 ± 3.9	13.0 ± 3.6			

#, Mann–Whitey U test; SD, standard deviation; Adj *p*-value, Benjamini–Hochberg correction sig. *p* < 0.05; **, Sig. at *p* < 0.01.

## Data Availability

Data generated and analysed for this study will be available from the corresponding author upon reasonable request.
